# Application of microorganisms towards synthesis of chiral terpenoid derivatives

**DOI:** 10.1007/s00253-012-4304-9

**Published:** 2012-07-31

**Authors:** Renata Kuriata-Adamusiak, Daniel Strub, Stanisław Lochyński

**Affiliations:** 1 Department of Bioorganic Chemistry, Faculty of Chemistry, Wrocław University of Technology, Wyb. Wyspiańskiego 27, 50–370 Wrocław, Poland; 2 Institute of Cosmetology, Wrocław College of Physiotherapy, Kościuszki 4, 50–038 Wrocław, Poland

**Keywords:** Biotransformations, Terpenoids, Microorganisms, Stereochemistry

## Abstract

Biotransformations are a standard tool of green chemistry and thus are following the rules of sustainable development. In this article, we describe the most common types of reactions conducted by microorganisms applied towards synthesis of chiral terpenoid derivatives. Potential applications of obtained products in various areas of industry and agriculture are shown. We also describe biological activity of presented compounds. Stereoselective hydroxylation, epoxidation, Baeyer–Villiger oxidation, stereo- and enantioselective reduction of ketones, and various kinetic resolutions carried out by bacteria and fungi have been reviewed. Mechanistic considerations regarding chemical and enzymatic reactions are presented. We also briefly describe modern approaches towards enhancing desired enzymatic activity in order to apply modified biocatalysts as an efficient tool and green alternative to chemical catalysts used in industry.

## Introduction

Terpenoids are group of compounds of natural origin biosynthesized from isopentenyl pyrophosphate (IPP) and dimethylallyl pyrophosphate (DMAPP). Structurally, they are saturated and unsaturated cyclic and aliphatic hydrocarbons with varying degrees of oxygenation, including alcohols, aldehydes, ketones, and carboxylic acids. Terpenoids can be divided into subclasses according to number of isoprene units in their structure. Condensation of IPP and DMAPP leads to geranyl pyrophosphate (GPP, C_10_) which is a precursor in biosynthesis of monoterpenoids and iridoids. Reaction between GPP and IPP gives farnesyl pyrophosphate (FPP, C_15_)–precursor in biosynthesis of sesquiterpenoids and sesquiterpenoid lactones. Condensation of two units of FPP leads to triterpenoids (C_30_). Among them are limonoids, cardenolides, quassinoids, cucrbitacins, saponins, and phytosterols. From terpenoids, we can also distinguish diterpenoids class (C_20_) which originate from geranylgeranyl pyrophosphate.

Terpenoids are applied in varying areas of human interest: flavor and fragrance industry–volatile monoterpenoids like citronellal, citral, geraniol, (−)-menthol; pharmaceutical industry–anticancer agent taxol; agriculture–iridoids and sesquiterpene lactones which are used as insect feeding deterrents (Walton and Brown 1999). Despite variety of applications and activity of terpenoids, there are attempts to modify their base structure to enhance their properties. This can be achieved by applying chemical synthesis or biotransformations. In many cases, organic synthesis is not a method of choice of structural modifications of complicated terpenoids due to side reactions, low regio- and enantioselectivity, low yields, and application of expensive catalysts comprising transition metal ions.

Microorganisms have been used for a long time in household and industry, where they play indispensable role. Their application extends to food processing as well as to catalysis of complicated chemical reactions.

Undisputed advantages of catalytic use of whole cells of microorganisms are their broad substrate specificity, possibility to act under mild conditions, and the fact that they do not require regeneration of cofactors comparing to isolated enzymes. Application of microorganisms is more economical than the use of enzymes but does not always result in similarly high enantioselectivity of catalyzed reactions (Nakamura et al. 2002; Beloqui et al. 2008). In order to minimize occurrence of side reactions and to enhance enantioselectivity manipulation of reaction conditions have been elaborated, to mention only stereochemical control as well as addition of organic solvents or enzyme inhibitors (Nakamura 1998).

Despite several disadvantages, microbial transformations are commonly used in organic synthesis towards preparation of homochiral compounds used in agriculture, pharmaceutical, cosmetic, and food industry. In our previous papers, we described microbiological methods of obtaining new terpenoid derivatives with biological activity such as odorants (Kuriata et al. 2010; Kuriata-Adamusiak et al. 2011) and insect-feeding deterrents (Wincza and Lochyński 2012).

In this paper, we have described five main reaction types that microorganisms can conduct on terpenes and terpenoids. They are stereoselective hydroxylation, epoxidation, Baeyer–Villiger oxidation, stereo- and enantioselective reduction of ketones, and kinetic resolution.

In the literature, we can find several reviews about biotransformations of terpenoids (de Carvalho and da Fonseca 2006; Ishida 2005; Bicas et al. 2009). De Carvalho and da Fonseca show many examples of biotransformations by different microorganisms resulting in terpenoid derivatives with mainly olfactory properties. Ishida describes biotranformations of terpenoids by mammals, microorganisms, and plant-cultured cells. His work is mainly focused on transformations carried out by mammals. The last one presents only bio-oxidation of terpenoid derivatives especially useful in flavor industry.

Our work is not focused on the only one type of the reaction which microorganisms can carry out on terpenoid derivatives. It can be considered as a brief review regarding most common and applicable reactions with description of organoleptic properties and biological activity. Moreover, we show the stereoselectivity of biocatalysts which is desired in many branches of industry. The goal of our work was to show background to intelligent research planning based on the type of the reaction with green alternative to toxic chemical catalysts. In addition, we describe modern approaches towards enhancing biocatalysts activity by site-directed mutagenesis and directed evolution. These methods may lead to development of procedures for large-scale preparation of industrially relevant terpenoids.

## Stereoselective hydroxylation

Hydroxylation reaction involves direct oxidation of C–H bond to corresponding alcohol. This transformation is difficult to achieve chemically due to inertness of C–H bonds of saturated hydrocarbons. Different strategies are employed to achieve chemo-, regio-, and enantioselectivity of hydroxylation. Chemical hydroxylations are often run in low substrate conversions to prevent overoxidations. Different oxidation systems show various selectivity towards primary, secondary, and tertiary C–H bonds. Exploiting directing groups that can coordinate to metal atoms of catalyst is the most favorable method of enhancing regioselectivity of hydroxylation. Additionally, stereoselectivity of these processes can be achieved with application of chiral auxiliaries (Baran 2009).

Various oxidants can be used to oxidize C–H bonds including transition metal catalysts [Pt(II)/Pt(IV) and Pd(II)/Pd(IV) systems] which are selective towards terminal C–H bonds (Neufeldt and Sanford 2012). For oxidations of secondary and tertiary C–H bonds in saturated compounds, dioxiranes are reagents of choice. Dioxiranes are strong oxidants and cannot be applied to hydroxylation of alkenes due to epoxide formation.

Transition metal catalysts are especially useful for synthesis of some steroids from triterpenoids. Mechanism of chemical hydroxylation of a triterpenoid *E*-lupanone oxime with palladium catalyst is a good example of this type of C–H bond activation and oxidation (Carr et al. 1988). *E*-Lupanone oxime consists of C=N–OH group which is directing sodium chloropalladate to complex with adjacent methyl group. Resulting dimer is complexed with pyridine and Pd–C bond is oxidized with lead(IV) acetate to form acylated product in 4α-methyl position.

Hydroxylation is the most extensively studied reaction catalyzed by whole cells of microorganisms. Enzymes responsible for these transformations belong to oxygenases family.

Oxygenases use molecular oxygen as an oxidant. They are cytosolic and transmembrane proteins and may lose their activity upon isolation (Holland and Weber 2000). This is the reason why hydroxylation is the least understood reaction conducted by microorganisms. Oxygenases performing hydroxylation reactions use only one oxygen atom to oxidize a substrate and are called monooxygenases. One of the most thoroughly studied monooxygenases are cytochromes P450 (Meunier et al. 2004). They are present in all branches of life. They consist of an Fe(III)-protoporphyrin-IX covalently linked to the protein by the sulfur atom of cysteine. Mechanism of enzymatic hydroxylation involves several oxidation-reduction processes of an iron atom in porphyrin binding site. Presence of a substrate in a binding site results in elimination of complexed water molecule. Next steps involve reduction of Fe(III) to Fe(II), activation in situ of oxygen molecule to form complexed peroxide radical which is subsequently reduced to peroxide anion. Two protonations of oxygen molecule in peroxo complex result in loss of water and formation of Fe(IV)^+°^ complex which is responsible for hydroxylation of a substrate R–H.

Hydroxylation allows synthesis of many drug precursors (Holland 1999) and is a useful alternative for preparation of optically active terpenoid compounds (Kołek et al. 2009).

**Fig. 1:**
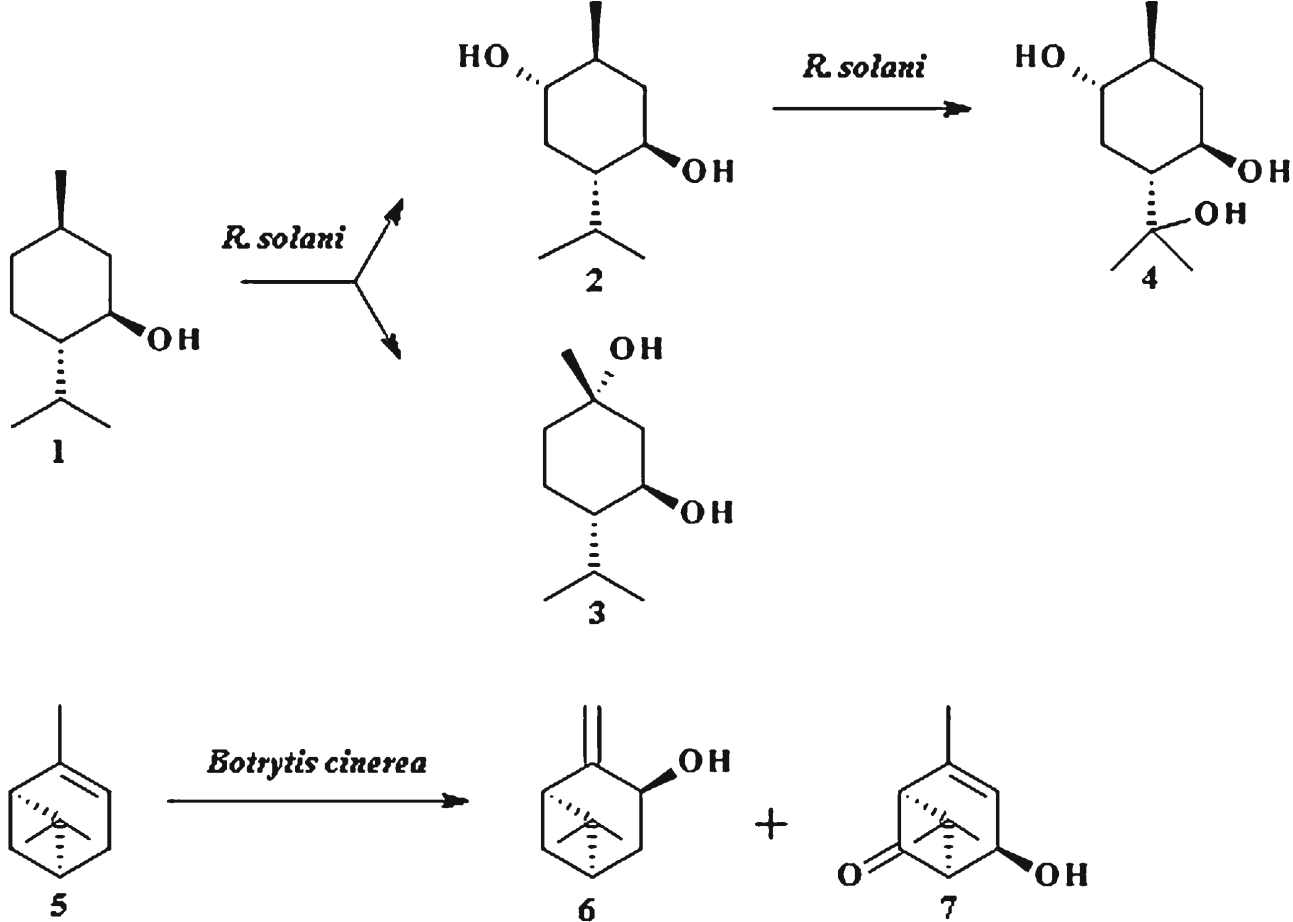
Biotransformation of (−)-menthol by *R. solani* and (−)-α-pinene by *B. cinerea*

An example of stereoselective hydroxylation of terpenoids is biotransformation of (−)-menthol 1 by 12 isolated species of *Rhizoctonia solani* (Fig. 1; Miyazawa et al. 2003). (−)-Menthol 1 is a constituent of mint oil; however, its production from natural sources is too low to cover all the needs. It is also possible to synthesize it from (+)-citronellal, thymol or myrcene, which are quite easily available substrates. (−)-Menthol is commonly used as a food, cosmetic, and pharmaceutical additive due to its pleasant flavor and fragrance. Biotransformations of 1 by *Aspergillus* species (Asakawa et al. 1991), *Penicillium* species (Esmaeili et al. 2009), and *Rhizoctania solani* (Miyazawa et al. 2002) are described in literature. For example, Miyazawa et al. have shown that three out of 12 species of *R. solani* produced: (−)-6-hydroxymenthol 2 (65.2 %), (+)-6,8-dihydroxymenthol 4 (32.4 %), and (−)-1-hydroxymenthol 3 (18.4 %) are intermediates for syntheses of compounds which can be applied as agents for control of snow blight disease. Corresponding acetates of alcohols 2 and 4 possess equal lytic activity against the fungus *Micronectriella nivalis* comparing to organocopper agent copper 8-hydroxyquinolate (Miyazawa et al. 2003).

The second example of microbial hydroxylation is biotransformation of (−)-α-pinene 5 by fungi (Fig. 1). Compound 5 is a major and important constituent of essential oils derived from plants. Microbial transformation of 5 by *Botrytis cinerea* resulted in obtaining (1*R*,3*S*,5*R*)-*trans*-*β*-pinen-3-ol 6 (10 %) and (1*S*,2*R*,5*S*)-*trans*-7-oxoverbenol 7 (16 %; Farooq et al. 2002). Activity of 6 and 7 was not elucidated but it would be interesting to evaluate fragrance and antifungal potential in regard to structural modification.

## Epoxidation

Oxidation of a double bond is a useful tool in organic synthesis. Epoxides are intermediates in preparation of biologically active molecules (Lochyński et al. 2002; Moniczewski et al. 2011) including aminoalcohols, diols and hydroxyazides. Most commonly used epoxidation reagents for preparation of racemic epoxides are hydrogen peroxide and peracids due to their low cost. High degree of polarization of peracids allows addition of electrophilic oxygen to an alkene and shifting proton simultaneously. Described reagents are not suitable for epoxidation of substrates comprising multiple double bonds in their structure due to lack of regioselectivity of peracids. In addition, if optically pure epoxides are desired, hydrolytic kinetic resolution of racemic oxiranes with Jacobsen’s catalyst must be employed. For chemical preparation of complex optically active epoxides transition, metal catalysts are employed including vanadyl acetylacetonate [VO(acac)_2_] (Itoh et al. 1979) and titanium isopropoxide [Ti(O*i*Pr)_4_] with salalen ligands (de Faveri et al. 2011). When alkene is sterically hindered in proximity of a double bond, dioxiranes can be applied for epoxidations with a fair to good stereoselectivity due to electrophilic attack on less hindered face of a substrate (Adam et al. 1999).

Biocatalytic oxidation of C=C bond is catalyzed by oxygenases and peroxidases, which results in production of epoxides with high enantiomeric and diastereomeric excess. Peroxidases use hydrogen peroxide as an oxidant, a process which decreases stability of forming epoxides for their further enzymatic transformations. Peroxidases are usually cytochrome P450 enzymes which contain heme prosthetic group. Iron atom is also coordinated with sulfur atom of protein’s cysteine. Peroxidases are able to incorporate hydrogen peroxide to form Fe(III)–hydroperoxide complex which performs attack on double bond of an alkene (de Maria et al. 2010). Oxygenases employ molecular oxygen, which is activated at the active site of the enzyme. Activation in situ results in fewer side reactions. Mechanism of epoxidation by monooxygenases is similar to hydroxylation. In this case, the active agent which reacts with substrate is also Fe(IV)^+°^ complex (Vaz et al. 1998).

Biotransformations of alkenes leading to epoxides require fast removal of products due to low stability of oxiranes in water solutions. In addition, high concentration of substrates and products may decrease enzyme activity. When designing high-scale biocatalytic epoxidations, it is necessary to adjust carefully reaction conditions in order to obtain higher yields (Li et al. 2002).

**Fig. 2:**
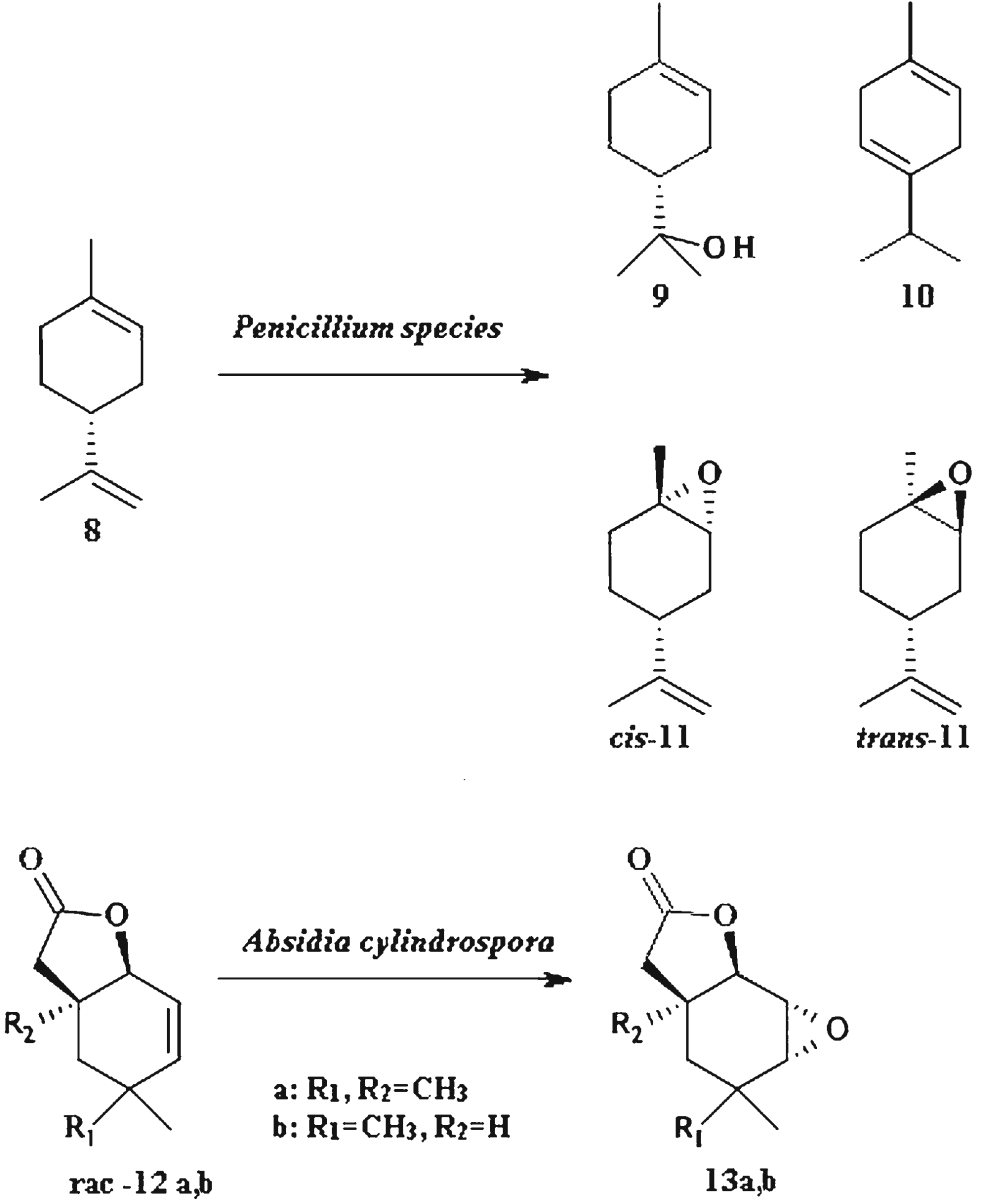
Biotransformation of (*R*)-(+)-limonene by *Penicillium* species and 4,4,6-trimethyl-9-oxabicyclo[4.3.0]non-2-en-8-one with *A. cylindrospora*

There are many literature examples describing preparation of terpenoid epoxides via biotransformations (Fraga et al. 2005). Over 60 fungi species were tested by Demyttenaere et al. for their capability of transforming (*R*)-(+)- and (*S*)-(−)-limonene, which yielded *trans* and *cis* epoxides using solid-phase microextraction as the monitoring technique (Fig. 2). Products of biotransformation of (*R*)-limonene 8 by *Penicillium digitatum* were mainly (*R*)-(+)-α-terpineol 9 and γ-terpinene 10. *cis*-Limonene oxide *cis*-11 and *trans*-limonene oxide *trans*-11 were the only side products in this reaction (Demyttenaere et al. 2001). Approaches to biosynthesize limonene oxides are relevant due to high repellent activity of *cis-*11 and *trans-*11. Limonene oxide is a major constituent of essential oil of the repellent plant *Lippia javanica* (Omolo et al. 2004). In addition, several international patents regarding application of limonene oxide as insect repellent were prepared.

Another example of microbial epoxidation is biotransformation of unsaturated bicyclic γ-lactone (Fig. 2). *Absidia cylindrospora* efficiently transforms 12a and 12b to corresponding *trans*-epoxylactones 13a and 13b where 13b was obtained with 100 % diastereomeric excess (Gładkowski et al. 2007). These compounds were not previously described in literature and there are no information regarding biological activity. Evaluation of antifeedant activity is advisable due to feeding deterrent potency of structurally similar epoxylactones (Dancewicz et al. 2011; Hollauf and Urban 1995).

Application of microorganisms to obtain epoxylactones is not a method of choice due to opening of oxirane ring followed by rearrangements in reaction conditions.

## Baeyer–Villiger oxidation

Baeyer–Villiger oxidation of linear and cyclic ketones to corresponding esters and lactones is an important reaction in synthesis of biologically relevant compounds (Mihovilovic et al. 2002; Kamerbeek et al. 2003). Nowadays, pharmaceutical industry demands efficient and environmentally friendly methods for synthesis of optically pure compounds including esters and lactones. The most popular reagents used for Baeyer–Villiger oxidation of ketones are organic peracids. Mechanism of chemical B–V transformation involves protonation of oxygen atom of ketone’s carbonyl group and subsequent nucleophilic attack of peroxy anion on positively charged carbon atom of carbonyl. Resulting complex undergoes structural rearrangements. The more substituted group adjacent to carbonyl group the better stabilization potential of positive charge it possesses. This property is responsible for higher migratory aptitude of tertiary carbon atoms over secondary and primary. At the final step, carbonyl group is deprotonated and final product is formed.

Microorganisms that produce Baeyer–Villiger monooxygenases (BVMO) are able to oxidize ketones with high regio- and stereospecifity. This family of enzymes contain flavin mononucleotide (FMN) or flavine adenine dinucleotide (FAD) as a cofactor. FAD or FMN undergoes protonation of nitrogen atom which allows activation of molecular oxygen to form FMN- or FAD-peroxide anion complex. Peroxide anion oxidizes ketone molecule and cofactor is regenerated with subsequent loss of water molecule (Kamerbeek et al. 2003). Microbial B–V oxidation is a good alternative to the reaction catalyzed by toxic organic peracids (Renz and Meunier 1999).

**Fig. 3:**
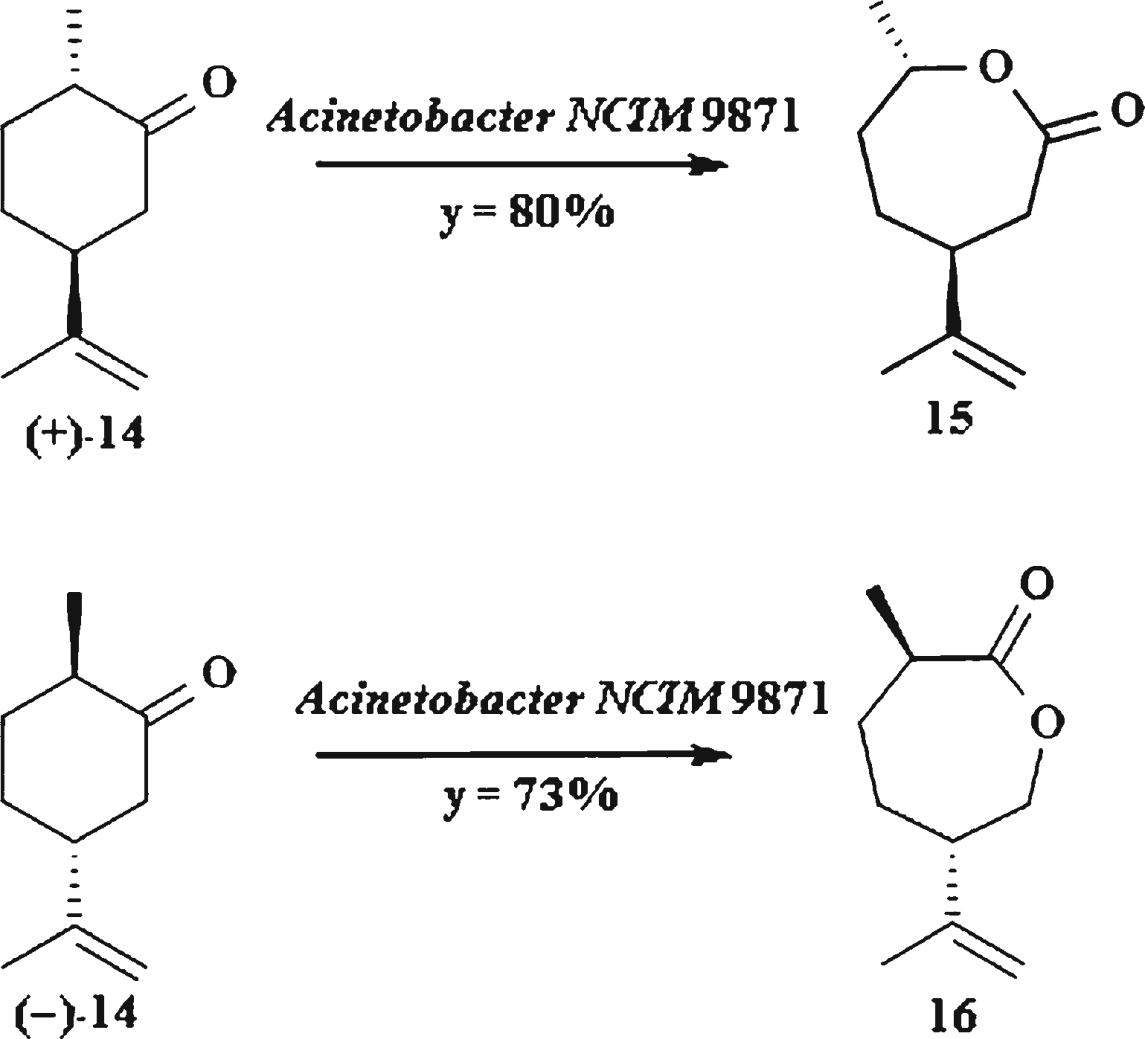
Biotransformation of (±)-dihydrocarvone by *A. calcoaceticus*

An interesting example of microbial Baeyer–Villiger oxidation is biotransformation of (±)-dihydrocarvone 14 by *Acinetobacter calcoaceticus*, resulting in two lactones (Fig. 3; Ottolina et al. 1996). Monooxygenase from this species exhibits regio- and enantiospecifity. (+)-Dihydrocarvone (+)**-**14 is transformed into lactone 15 with regard to the higher migratory aptitude of tertiary carbon atom adjacent to the carbonyl group. Biotransformation of this terpene by *A. calcoaceticus* leads to migration of secondary carbon atom during oxidation resulting in lactone 16. This compound is an intermediate in synthesis of (3*S*,6*R*)-3-methyl-6-(1-methylethenyl)-9-decen-1-yl acetate which is an attractant for male *Aonidiella aurantii*—citrus fruits pest and can be applied with combination with other pest control agents (Anderson et al. 1980).

**Fig. 4:**
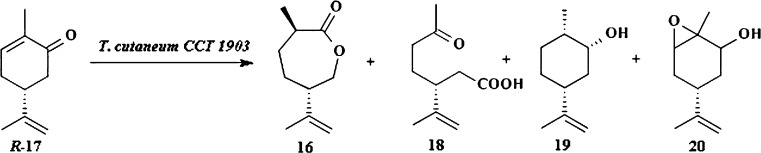
Biotransformation of (4*R*)-(−)-carvone by *T. cutaneum* CCT 1903

The next example of whole cells Baeyer–Villiger oxidation is biotransformation of (4*R*)-(−)-carvone *R*-17 by yeast *Trichosporum cutaneum* CCT 1903 (Fig. 4; Pinheiro and Marsaioli 2007). The main product is hydrogenated lactone 16 (31 %) and side products–ketoacid **18** (5 %), dihydrocarveol 19 (3.8 %), and epoxydihydrocarveol 20 (2.2 %).

Biocatalytic Baeyer–Villiger oxidation can obviously be performed also by isolated enzymes. The most common sources of isolated BVMO are species of *Acinetobacter* (Secundo et al. 1993), *Nocardia* (Norris and Trudgill 1971), and *Pseudomonas* (Alphand et al. 1996).

According to the literature (Roberts and Wan 1998; Alphand et al. 1996), it is not possible to state which type of biocatalyst is better. In the case of microbial transformations, we can develop single-step procedures for preparative scale synthesis of chiral terpenoids. In addition, sometimes the use of microorganisms results in formation of an unusual products as it was observed in the case of compound 16. On the other hand, for some substrates, application of isolated monooxygenases is more suitable due to higher yields and stereopurity of synthesized products.

## Stereo- and enantioselective reduction of ketones

Reduction of ketones to corresponding alcohols is the most commonly studied transformation catalyzed by whole cells of microorganisms. Biocatalysts are good alternative to ruthenium (Zanotti-Gerosa et al. 2005), catecholborane (Evans and Hoveyda 1990), or oxazaborolidine reagents (Hirao et al. 1981) popularly used for stereoselective reduction. These reagents use specific interactions between catalysts core and carbonyl group.

Application of whole cells of microorganisms is more convenient method for stereoselective reduction comparing to isolated enzymes due to lack of necessity for using additional system for cofactors regeneration. Many species of fungi and yeast were used for stereoselective reduction of prochiral ketones to optically pure alcohols. Stereoselectivity of ketone reduction depends on specific interactions between enzyme and substrate. This reaction is mostly catalyzed by alcohol dehydrogenase (ADH). These biomolecules are nonheme reductive/oxidative enzymes. ADH’s perform hydrogen transfer reaction in presence of coenzyme which acts as a hydrogen donor/acceptor (Naik et al. 2012). The most extensively studied ADH’s are medium-chain alcohol dehydrogenases which usually contain two zinc ions (Ying and Ma 2011). The catalytic zinc ion interacts with three ADH’s ligands–one histidine and two cysteine residues and a water molecule. Structural zinc site is highly conserved for all classes of alcohol dehydrogenase family and mostly consists of Zn ion bound to four cysteine residues (Auld and Bergman 2008). Most microorganisms produce several dehydrogenases which are able to oxidize different hydroxyl groups and reduce different carbonyl groups (Carballeira et al. 2009). Most of the applicable alcohol dehydrogenases prefer small, nonpolar, and non-ionisable ketones as substrates (Zhu et al. 2006).

**Fig. 5:**
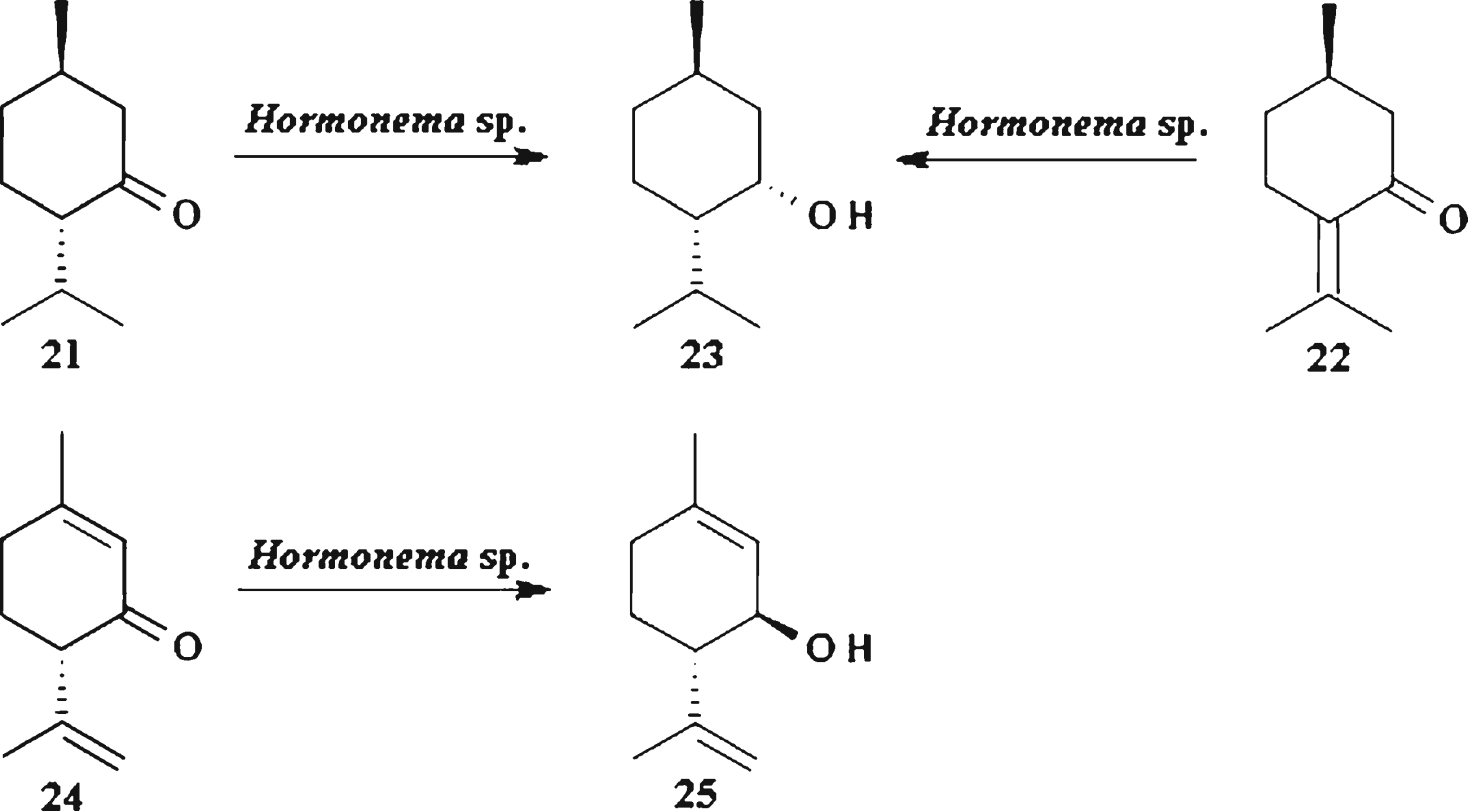
Biotransformations by *Hormonema* sp. UOFS Y-0067

An example of selective bioreduction of terpenes are biotransformations of (−)-menthone 21 and (+)-pulegone 22 to (1*R*,3*S*,4*S*)-(+)-neomenthol 23 (Fig. 5). Biocatalyst used for this transformation was *Hormonema* sp. UOFS Y-0067 (van Dyk et al. 1998). Mironowicz and Siewinski (1986) reported that cells of *Rhodotorula mucilaginosa* immobilized on polyacrylamide gel can also be applied for stereoselective reduction of 21 to 23. (+)-Neomenthol 23 possesses antifungal activity against *Fusarium verticillioides* which causes *bakanae* disease in rice seedlings and *Colletotrichum gloeosporioides*–plants pathogenic fungus infesting apple and mango (Dambolena et al. 2010; Nidiry 2003).

Yeast *Hormonema* sp. UOFS Y-0067 was also applied for selective reduction of (4*S*)-isopiperitenone 24. Product of this biotransformation was exclusively optically pure (3*R*,4*S)*-isopiperitenol 25 (Fig. 5). Isopiperitenol 25 is an intermediate in preparation of (−)-menthol 1 which production from natural sources is not sufficient as was mentioned before (Serra et al. 2003). It is worth noting that configuration of alcohol 25 is opposite to the alcohol 23 despite the fact that stereochemistry of carbon atom C-4 is the same in both substrates.

Another example of selective bioreduction is biotransformation of (4*R*)-carvone *R*-17. Several microorganisms were screened by Carballeira et al. for ability to perform selective reduction of different ketones and the best results were achieved using species of *Gongronella butleri*, *Diplogelasinospora grovesii*, and *Schizosaccharomyces octosporus* (Carballeira et al. 2004).

**Fig. 6:**
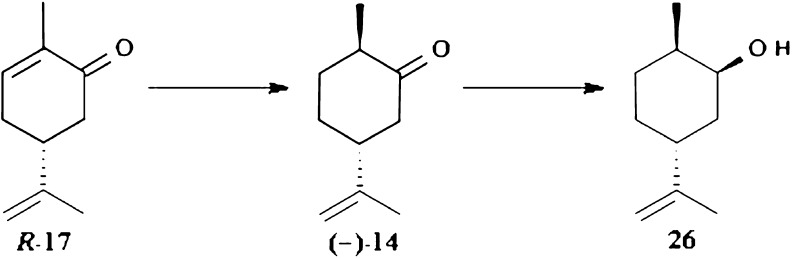
Reduction of (4*R*)-carvone by *G. butleri*, *D. grovesii*, and *S. octosporus*

In case of biotransformation of (4*R*)-carvone the first step was hydrogenation of *R*-17 to (1*R*,4*R*)-dihydrocarvone (−)-14 and subsequent stereoselective reduction to (1*R*,2*S*,4*R*)-dihydrocarveol 26 (Fig. 6). In addition, when *G. butleri* had been used as biocatalysts, a small amount of side products were present. Dihydrocarveol 26 is a flavoring food additive so environmentally friendly method of its production would be desirable.

**Fig. 7:**
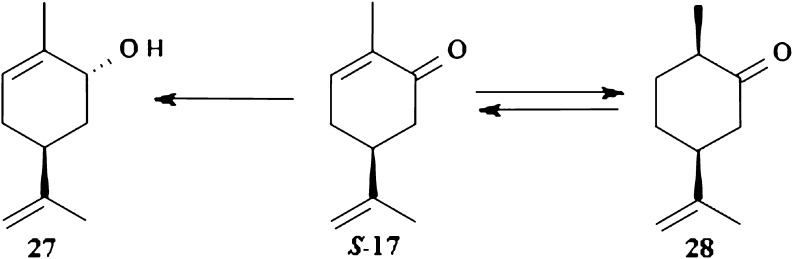
Biotransformation of (4*S*)-carvone by *G. butleri*, *D. grovesii*, and *S. octosporus*

The same biocatalysts were used for bioreduction of second isomer of carvone–(4*S*)-carvone *S*-17. Biotransformation by *D. grovesii* resulted in the formation of allyl alcohol 27 which possesses fragrance resembling that of mint and caraway. It is used in cosmetic industry and as flavor additive in food industry. The same product was obtained in case of using *G. butleri* and *S. octosporus.* In these cases, they contained traces of additional compounds. (4*S*)-Carvone was also hydrogenated to (1*R*,4*S*)-dihydrocarvone 28 in conditions similar to those applied to *R*-isomer but in this case reduction did not occur. It is postulated that unstable primary reaction product (1*R*,4*S*)-dihydrocarvone is oxidized to (4*S*)-carvone which is subsequently reduced to secondary stable product (2*R*,4*S*)-carveol 27 (Fig. 7). Additionally, the factor determining direction of the reaction pathway is stereochemistry at carbon atom C-4.

**Fig. 8:**
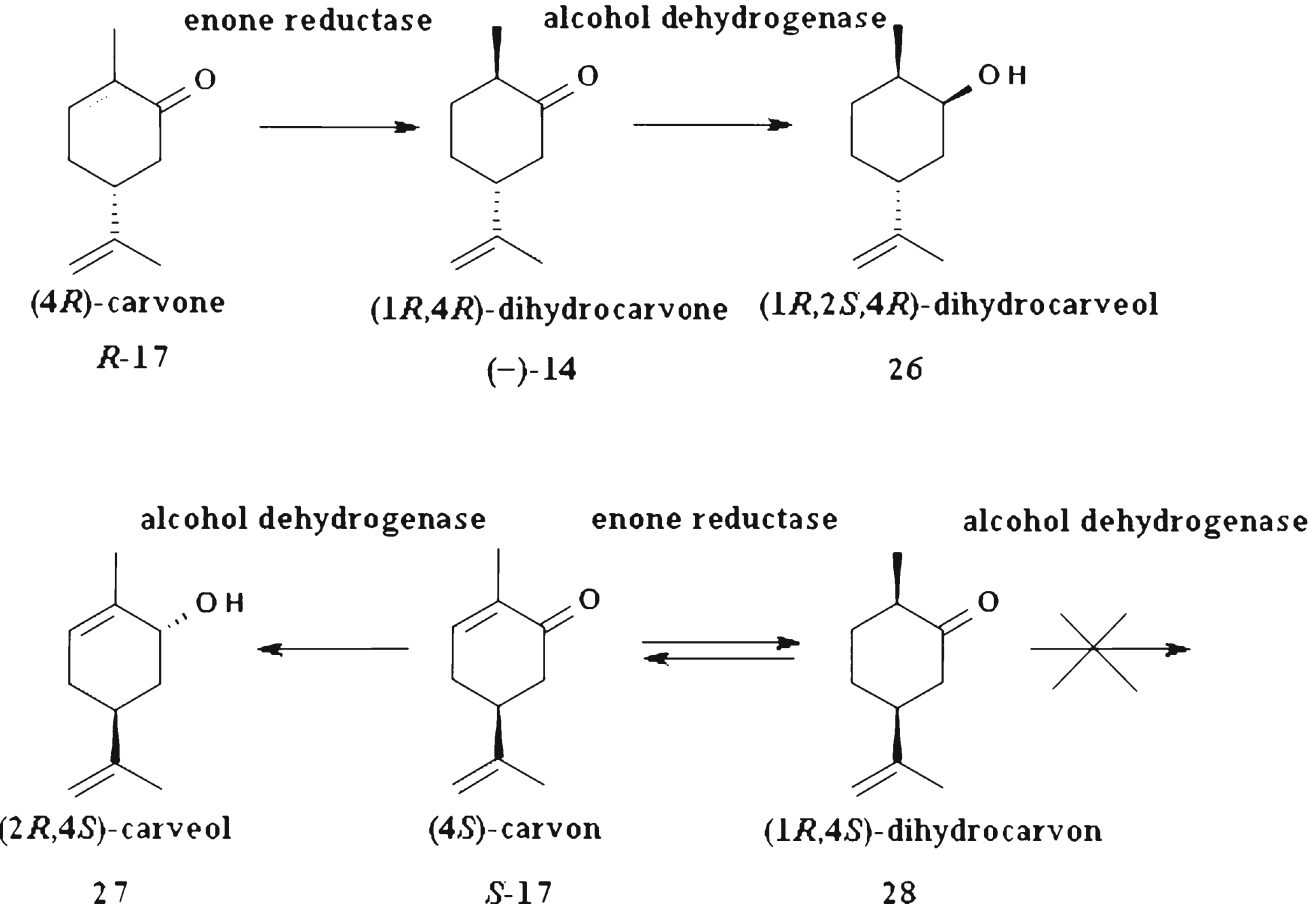
Profile of reduction of carvone stereoisomers with selected biocatalysts

Summarizing carvone bioreduction, it is seen that reaction pathway is different for each enantiomer. For both isomers the most interesting catalyst was *D. grovesii* which gives maximum yield of alcohol and no side products. Stereochemistry at C-4 carbon atom is a factor determining direction of the reaction pathway due to action of two main enzymes involved in transformations (Fig. 8):

1. enone reductase which in both cases catalyzes reaction to (1*R*,4*R* or *S*)-dihydrocarvone (Faber 2000)
2. alcohol dehydrogenase which reduces only one isomer of dihydrocarvone-(1*R*,4*R*)-dihydrocarvone to dihydrocarveol. It also catalyzes exclusively reduction of carbonyl group of (4*S*)-carvone to (2*R*,4*S*)-carveol. The same conclusions were made by van Dyk et al. (1998).

## Kinetic resolution

Nowadays, searching for the new efficient methods for synthesis of optically pure chiral building blocks is of great interest to industrial and academic laboratories (de Carvalho et al. 2002). In the past decade, advances have been made towards asymmetric synthesis but kinetic resolution of racemic mixtures is still the most often applied method for preparation of optically pure compounds in industry (Robinson and Bull 2003).

**Fig. 9:**
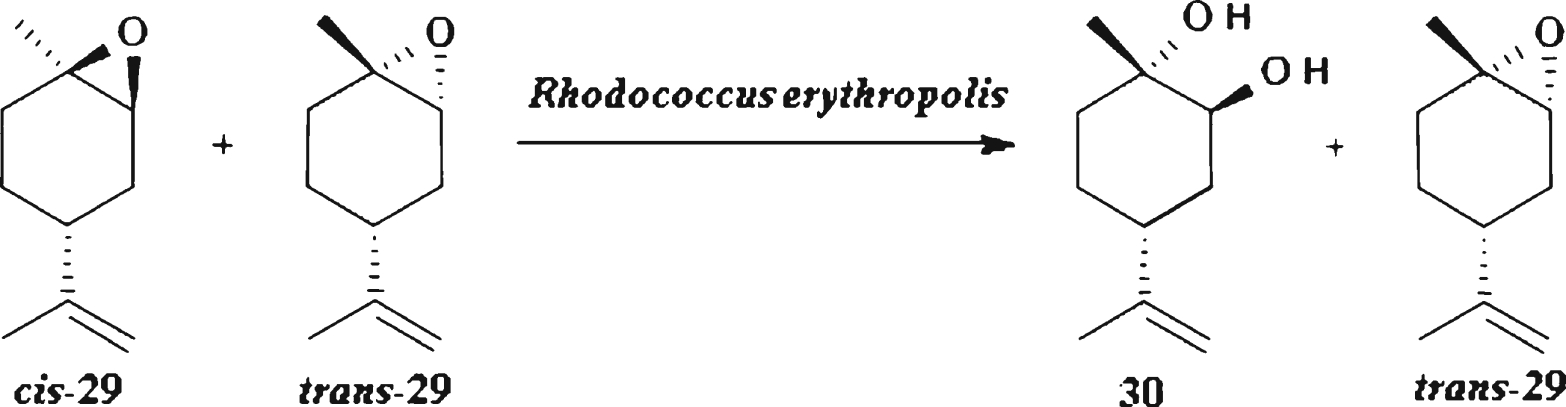
Kinetic resolution of (+)-(1*R*,2*S*,4*R*)-1,2-epoxylimonene and (+)-(1*S*,2*R*,4*R*)-1,2-epoxylimonene using *R. erythropolis* DCL14 as a biocatalyst

An example of efficient bioresolution of terpenoid substrate is biotransformation of (+)-1,2-epoxylimonene used in form of mixture of isomers *cis*-29 and *trans*-29 in a ratio of 42.2:55.7 by applying *Rhodococcus erythropolis* DCL14 as a biocatalyst (Fig. 9; van der Werf et al. 1999a, b). As a result of catalytic action of 1,2-epoxylimonene hydrolase optically pure (+)-(1*S*,2*S*,4*R*)-limonene-1,2-diol 30 and unreacted (+)-(1*S*,2*R*,4*R*)-1,2-epoxylimonene *trans*-29 were obtained.

**Fig. 10:**
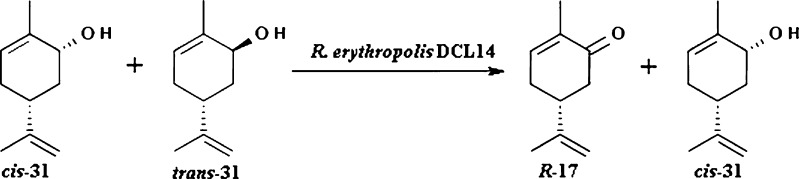
Kinetic resolution of (−)-(4*R*,6*R*)-carveol and (−)-(4*R*,6*S*)-carveol with *R. erythropolis* DCL14

The same microorganism was used to resolve diastereomeric mixture of carveol 32 by selective oxidation. Biotransformation of mixture of (−)-*cis*-31 and (−)-*trans*-31 in a ratio of 38:62 by *R. erythropolis* DCL14 revealed high stereoselectivity of dehydrogenase. As a result, two optically active products were obtained: (−)-(*R*)-carvone *R*-17 and (−)-*cis*-carveol 31 (Fig. 10; Tecelao et al. 2001; van der Werf et al. 1999a, b).

## Future prospects

Despite intensive studies on whole cells biotransformations of terpenoids, not all issues regarding stability and selectivity have been resolved. In case of water-sensitive compounds, microbial transformations are not a method of choice due to inability of maintaining water-free conditions. The solution is to carry on transformations with purified enzymes in organic solvents. The major problem of applying microorganisms for biotransformations might be low selectivity like in case of hydrolases. Different enzymes belonging to the same family may have various regio- or stereoselectivity towards transformed compound. Modern studies concentrate on genetic modification of microorganisms towards overexpression and mutations of enzymes useful in biotransformations (Schulz 2007). Wild-type enzymes usually are not well adapted to non-natural substrates which may lead to low conversions or low stereoselectivity. This issue may be overcome by biocatalyst optimization. Genetic screening of microorganisms or screening of metagenomic libraries are useful tools for discovery of new naturally evolved enzymes which may exhibit better properties regarding biotransformation of compound of our interest (Fraaije et al. 2005; Daniel 2004). An alternative method is enzyme engineering of known biocatalysts using site-directed mutagenesis or directed evolution. The first approach is more elegant, gives better results, and often is supported by bioinformatic studies but requires extensive knowledge about enzyme’s structure. On the other hand, directed evolution is generally based on creation of library of mutated genes coding biocatalyst, proper plasmid preparation, transformation, and incubation with substrate. A good example is mini-evolution of cyclopentanone monooxygenase which allowed transforming 4-methylcyclohexanone into corresponding lactone with 92 % enantiomeric excess compared to the 46 % *ee* achieved with wild type cyclohexanone monooxygenase (Clouthier et al. 2006).

Wild type or recombined enzymes must be overexpressed in a proper host to be considered as a biocatalyst for large-scale processes. The first kilogram scale Baeyer–Villiger oxidation was reported by Hilker et al. (2005) where racemic bicyclo[3.2.0]hept-2-en-6-one was converted into two lactones with excellent *ee*’s: >98 and >99 %.

Despite good results, commenced studies over large-scale microbial transformations are focused generally on biotransformations of small, nonpolar molecules. The challenge is to develop system suitable for carrying out biotransformation of larger, more polar, and multifunctional compounds.

## Conclusion

Reactions conducted by various microorganisms using terpenoid derivatives as substrates and reviewed in this paper indicate vast possibilities for the production of optically pure compounds using this type of biocatalysts. Optical purity is essential for pharmaceutical and fragrance industry due to possible differences in organoleptic properties and biological activity of isomers. The diversity of microbial activity demonstrates that for each type of reaction it is possible to select proper microorganism that will efficiently and selectively convert substrates into desired products. Modern studies are focused on modification of microorganisms towards overexpression of enzymes of our interest and to enhance desired activity of known enzymes by directed evolution. These studies are particularly valuable to industry due to possibility of carrying out large scale biotransformations.
